# Association between general practice characteristics and use of out-of-hours GP cooperatives

**DOI:** 10.1186/s12875-015-0266-1

**Published:** 2015-05-01

**Authors:** Marleen Smits, Yvonne Peters, Sanne Broers, Ellen Keizer, Michel Wensing, Paul Giesen

**Affiliations:** Radboud university medical center, Radboud Institute for Health Sciences, IQ healthcare, PO Box 9101, IQ healthcare 114 6500 HB, Nijmegen, The Netherlands

**Keywords:** Health Services Accessibility, Primary Health Care, Emergency Medical Services, After-Hours Care

## Abstract

**Background:**

The use of out-of-hours healthcare services for non-urgent health problems is believed to be related to the organisation of daytime primary care but insight into underlying mechanisms is limited. Our objective was to examine the association between daytime general practice characteristics and the use of out-of-hours care GP cooperatives.

**Methods:**

A cross-sectional observational study in 100 general practices in the Netherlands, connected to five GP cooperatives. In each GP cooperative, we took a purposeful sample of the 10 general practices with the highest use of out-of-hours care and the 10 practices with the lowest use.

Practice and population characteristics were obtained by questionnaires, interviews, data extraction from patient registration systems and telephone accessibility measurements. To examine which aspects of practice organisation were associated with patients’ use of out-of-hours care, we performed logistic regression analyses (low versus high out-of-hours care use), correcting for population characteristics.

**Results:**

The mean out-of-hours care use in the high use group of general practices was 1.8 times higher than in the low use group. Day time primary care practices with more young children and foreigners in their patient populations and with a shorter distance to the GP cooperative had higher out-of-hours primary care use. In addition, longer telephone waiting times and lower personal availability for palliative patients in daily practice were associated with higher use of out-of-hours care. Moreover, out-of-hours care use was higher when practices performed more diagnostic tests and therapeutic procedures and had more assistant employment hours per 1000 patients.

Several other aspects of practice management showed some non-significant trends: high utilising general practices tended to have longer waiting times for non-urgent appointments, lower availability of a telephone consulting hour, lower availability for consultations after 5 p.m., and less frequent holiday openings.

**Conclusions:**

Besides patient population characteristics, organisational characteristics of general practices are associated with lower use of out-of-hours care. Improving accessibility and availability of day time primary day care might be a potential effective way to improve the efficient use of out-of-hours care services.

## Background

Out-of-hours healthcare services are confronted with large numbers of low-urgency encounters with patients who could have waited until the opening hours of the day time general practice. Low-urgency contacts are contacts that could be postponed until the next day without increased risks. These low-urgency contacts contribute to the overcrowding crisis of hospital emergency departments (ED) [1-3] and to the high utilisation rates of other out-of-hours emergency medical services, such as general practitioner (GP) cooperatives in the Netherlands [4,5] (Table 1). Unnecessary use of out-of-hours care services contributes to inefficient use of care resources as contacts during out-of-hours are more expensive than during office hours [6,7]. Improved accessibility and availability of primary care during day time may reduce patients’ use of out-of-hours care by directing patient flows to general practices which are open during day time.

**Table 1:** **Features of general practitioner (GP) cooperatives in the Netherlands** [1]

**Theme**	**Feature**
General	Out-of-hours primary care is provided by large-scale general practitioner (GP) cooperatives
	Participation of 50–250 GPs per cooperative with a mean of 4 hours on call per week
	Circa 125 GP cooperatives in the Netherlands
	Population of 100,000 to 500,000 patients
	Out-of-hours defined as daily from 5 p.m. to 8 a.m. and the entire weekend
Location	GP cooperative usually situated in or near a hospital
	Distance of patients to GP cooperative maximally 30 km
Accessibility	Access via a single regional telephone number, meaning the first contact mostly is with a triage nurse (only 5-10% walk in without a call in advance)
	Telephone triage by nurses supervised by GPs: contacts are divided into telephone advice, centre consult, or GP home visit
Facilities	Drivers in identifiable GP cars that are fully equipped (e.g. oxygen, intra venous drip equipment, automated external defibrillator, medication)
	Information and communication technology (ICT) support including electronic patient files, online connection to the GP car, and sometimes connection with the electronic medical record in the GP daily practice

There is substantial variation in the use of out-of-hours GP cooperatives across patient populations of different general practices, as information from local GP cooperative registration systems suggests. The differential use of out-of-hours care may reflect differences in healthcare needs and patient behaviours. For instance, having many chronically ill patients in the practice implies higher use of healthcare. Likewise, it is known that parents of young children [8], foreigners [9,10], patients with low socioeconomic status [11-13] and patients living close to the service [12,14] relatively often contact acute care services, such as the ED and GP cooperative. However, the differential use of out-of-hours care could also be caused by variation in accessibility and availability of general practices [15]. Difficulties getting through to an assistant on the phone could result in patients contacting the national alarm number (112) or waiting until an out-of-hours care service is open. Poor availability for appointments could be a reason for patients to use other health care services than their own general practice. There are studies on the relation between access to primary care and acute care use, but most are based on patient self-reports [9,16-23]. The aim of this study was to examine which aspects of the organisation of day time primary care are related to the use of out-of-hours GP cooperatives. This knowledge is relevant for many countries who try to strengthen primary day time care.

## Methods

### Design and setting

We performed a cross-sectional observational study in a sample of 100 general practices, which were connected to five out-of-hours GP cooperatives, spread across the Netherlands. In each of the five GP cooperatives, we took a purposeful sample of the 10 general practices with patient populations with the highest out-of-hours care use and the 10 practices with the lowest use. One cooperative erroneously selected 11 high utilising practices and nine low utilising practices, resulting in a total of 51 practices in the high use group and 49 in the low use group. The selection of practices for the high and low use groups was thus performed at the level of the GP cooperative; the utilisation of a practice was relatively high or low compared to the other general practices that were connected to a specific GP cooperative (this explains the overlapping scores of the groups). Out-of-hours care use was defined as the number of contacts with the GP cooperative per 1000 patients, irrespective of the urgency of the contacts, and including telephone consultations, consultations at the cooperative and home visits. The GP cooperative is open daily from 5 p.m. to 8 a.m. and the entire weekend (see Table 1).

### Measures

All 100 general practices were visited by researchers, who used a combination of data collection instruments: a structured questionnaire for one practice assistant or GP, interviews with the GP(s), data extractions from the electronic information system and a telephone waiting time measurement.

Data extracted from the electronic system were practice size, number of contacts in one year, number of diagnostic tests and therapeutic interventions in one year and age distribution of patient population. We did not obtain data on patient level, only aggregated data on practice level. Telephone accessibility was measured by calling each practice 10 times, following a schedule which included all days of the week and all hours of the day, spread over a period of three weeks. The waiting time to personal contact with the assistant was calculated (maximum: 10 minutes). Data on two patient population characteristics -distance to the GP cooperative and proportion of foreign patients- were derived from Statistics Netherlands, based on the four-digit postal code of the general practice. All other data were gathered by means of a questionnaire for the assistant and interview with the GP(s).

The inclusion of themes in the measurement instruments was based on literature and expert consultation rounds. The instruments were pilot tested in 10 general practices. The study was double blinded: during the data collection period, neither the researchers nor the assistants/GPs were given information about the utilisation rate. GPs in the Netherlands do not have to record or account for their patients’ use of out of hours care.

Data were collected between August 2011 and November 2012. The out-of-hours care utilisation number was based on one calendar year (2010 in two GP cooperatives, 2011 in three cooperatives). The patient and practice characteristics related to the same period, except for the telephone accessibility measurement, which was performed eight to eleven months afterwards.

### Statistical analysis

General practices were the unit of analysis, because the utilisation scores were registered at that level. In case of general practices with more than one GP (N = 25), we included all GPs and averaged the scores on the GP characteristics within that practice (i.e. age, years of employment, work satisfaction, experienced workload). Frequencies or group means with ranges are presented for the group of general practices with low use at the GP cooperative and the group with high use. Numbers of contacts, medical procedures and employment days were transformed into numbers per 1000 patients.

The dependent variable in our analyses was dichotomous: low versus high use of out-of-hours care. Independent variables were patient population characteristics and general practice characteristics. Differences in population characteristics between the low and high use groups were examined with T-tests. We examined differences in practice characteristics using logistic regression analyses, correcting for three patient characteristics: 1) geographical distance from the general practice to the GP cooperative, 2) percentage of patients aged 0 to 4 years, and 3) percentage of foreigners. Odds ratios with 95% confidence intervals are reported. Differences were considered statistically significant at p < 0.05. Data were analysed using IBM SPSS 20.

### Ethics

The research ethics committee of the Radboud university medical center stated that the study does not fall within the remit of the Medical Research Involving Human Subjects Act (WMO). Therefore, informed consent was not needed according to Dutch law.

## Results

### Sample characteristics

The mean number of patients per general practice was 2485 (range 464 to 4556). The out-of-hours care use per practice in the low use group varied from 94 to 333 contacts per 1000 patients per year. It varied from 277 to 539 in the high use group. The mean out-of-hours care use in the high use group was 1.8 times higher than in the low use group (369 versus 204; p < 0.001). Other characteristics of the patient populations and general practices are shown in the first two data columns of Table 2 and Table 3.

**Table 2:** **Patient population characteristics of the general practices and association with out-of-hours care use**

**Variable**	**Low use group**	**High use group**
	**Mean (range)**	**Mean (range)**
Percentage of foreigners^a^	9.8 (1.6-26.4)	27.7 (3.8-82.2)**
Percentage of patients aged 0 to 4 years	4.8 (2.5-9.7)	5.6 (3.0-9.9)*
Distance from general practice to out-of-hours GP cooperative (kilometers)	13.4 (3.0-30.8)	3.4 (0.9-11.0)**

^a^People with at least one parent born outside the Netherlands.

*p < 0.01, **p < 0.001.

**Table 3:** **Practice characteristics and association with out-of-hours primary care use, adjusted for patient characteristics**

**Variable**	**Low use group**	**High use group**	**Odds ratio** ^**a**^
	**N (%)**	**N (%)**	**(95% CI)**
**Telephone accessibility**			
Waiting time on telephone (minutes)			
Mean (range)	1.04 (0–10)	1.97 (0–10)	1.26 (1.09-1.46)**
Median (range)***	0.41 (0–10)	0.88 (0–10)	
Telephone contact with assistant^b^ possible (no answering machine) (hours a week)			
Mean (range)	36.1 (9–45)	35.3 (14–45)	1.06 (0.97-1.15)
Telephone lines (per 1000 pts)			
Mean (range)	1.3 (0.3-4.6)	1.4 (0.4-6.4)	1.51 (0.44-5.25)
**Availability for consultations**			
Waiting time for non urgent consultations (days)			
Mean (range)	0.76 (0–5)	1.02 (0–5)	1.54 (0.67-3.53)
Consultation after 5 p.m. possible	14 (28.6)	8 (15.7)	0.42 (0.08-2.05)
Has telephone consulting hour	31 (63.3)	23 (45.1)	0.26 (0.05-1.34)
Open during holidays (with replacement)	24 (54.5)	15 (31.3)	0.42 (0.08-2.33)
Personally available for palliative patients (including out-of-hours)	41 (83.7)	25 (49.0)	0.22 (0.06-0.76)*
Clinician-hours (per 1000 pts)			
Mean (range)	11.1 (2.0-30.2)	12.6 (5.1-24.1)	0.98 (0.81-1.17)
**GP characteristics**			
Age (years)			
Mean (range)	51.9 (34–63)	53.6 (36–65)	1.00 (0.89-1.12)
Gender: female	6 (12.2)	9 (17.6)	2.38 (0.19-30.07)
Years of employment as GP			
Mean (range)	19.8 (5–34)	21.6 (3–39)	1.00 (0.92-1.09)
Work satisfaction (score 1–10; 1 = low;10 = high)			
Mean (range)	7.8 (7–9)	8.0 (4–10)	2.41 (0.80-7.23)
High experienced workload	26 (53.1)	38 (74.5)	2.18 (0.40-11.90)
Speak to patients with unnecessary out-of-hours contacts	9 (18.8)	13 (25.5)	1.29 (0.21-7.86)
**Practice organisation**			
Practice type: solo practice	32 (65.3)	27 (52.9)	0.95 (0.19-4.85)
Practice size (number of pts)			
Mean (range)	2459 (464–4103)	2511 (1295–4556)	1.00 (1.00-1.00)
Training in telephone triage for assistants^b^	8 (16.7)	18 (35.3)	1.87 (0.39-8.98)
Number of contacts (per pt)^c^			
Mean (range)	4.4 (1.7-10.7)	5.2 (2.3-8.4)	1.44 (0.85-2.44)
Number of diagnostic tests and therapeutic procedures (per 1000 pts)^d^			
Mean (range)	74.4 (5.1-304.8)	119.6 (4.4-935.4)	1.02 (1.01-1.04)**
**Presence of professionals** (half days a week, per 1000 pts)			
GPs			
Mean (range)	4.1 (2.1-8.8)	4.2 (1.9-10.5)	1.34 (0.66-2.75)
Assistants^b^			
Mean (range)	4.9 (2.1-14.4)	5.5 (2.7-11.0)	1.71 (1.07-2.73)*
Nurse practitioners mental health			
Mean (range)	0.2 (0.0-5.2)	0.3 (0.0-4.2)	4.61 (0.35-61.24)
Nurse practitioners somatics			
Mean (range)	1.1 (0.0-5.2)	1.6 (0.0-4.7)	2.07 (0.99-4.32)
Physicians in training			
Mean (range)	0.5 (0.0-3.1)	0.5 (0.0-3.9)	0.94 (0.38-2.40)

*p < 0.05; **p < 0.01; ***p < 0.001.

^a^Odds ratios adjusted for: 1) geographical distance from the general practice to the GP cooperative, 2) percentage of patients aged 0 to 4 years, and 3) percentage of foreigners.

^b^Assistants in Dutch general practices have administrative tasks, provide patient information, perform (telephone) triage and perform simple medical procedures (such as suturing, bandaging, giving injections, providing first aid and monitoring diabetic patients).

^c^Sum of telephone contacts, practice consultations, and home visits.

^d^Sum of six medical procedures, i.e. spirometry, electrocardiography, Doppler test, taping, intrauterine spiral, surgery.

### Patient characteristics and out-of-hours care use

General practices in the high use group had a higher mean percentage of foreigners (27.7%) and children in the age category 0 to 4 years (5.6%) compared to practices in the low use group (9.8% and 4.8%, respectively). Furthermore, high use practices were located at a shorter mean distance from the GP cooperative (3.4 km) than low use practices (13.4 km) (Table 2).

### Telephone accessibility and out-of-hours care use

The mean and median telephone waiting times were higher in the group of high use practices (mean: 1.97 minutes; median: 0.88 minutes) than in the group of low use practices (mean: 1.04 minutes; median: 0.41 minutes). The number of telephone lines and number of personal telephone contact hours were not related to the use of out-of-hours care (Table 3).

### Availability for consultations and out-of-hours care use

GPs in the high use group were less often personally available for palliative patients (49%) compared to GPs in the low use group (83.7%). In addition, several non-significant trends were found for the high use group indicating worse availability as compared to the low use group: longer waiting times for non-urgent consultations (1.02 versus 0.78 days), lower availability for consultations after 5 p.m. (15.7% versus 28.6%), lower availability of a telephone consulting hour (45.1% versus 63.3%), and less frequent holiday openings (31.3% versus 54.5%) (Table 3).

### GP characteristics and out-of-hours care use

GPs in the high use group did not differ from GPs in the low use group in age, sex, years of employment, work satisfaction, and talking to patients about unnecessary use of the GP cooperative. However, GPs in the high use group performed more therapeutic tests and diagnostic procedures per 1000 patients (119.6) than GPs in the low use group (74.4). Moreover, GPs in the high use group reported experiencing a higher workload (74.5% versus 53.1%), although this was not a significant difference (Table 3).

### Practice organisation and out-of-hours care use

Practices in the high use group had on average 5.2 contacts per patient per year, compared to 4.4 contacts in practices in the low use group (not statistical significant) (Table 3). The difference in the mean number of contacts per practice between the two groups was 1768 contacts, which is 13.9% of the contacts of practices in the low utilisation group and 16.1% of the contacts of practices in the high utilisation group (not in table). There were more assistant hours scheduled in the high use group (5.2 half days a week) than in the low use group (4.8 half days a week). There were no differences between the groups with regard to presence of GPs, nurse practitioners, and physicians in training, practice type, and triage training (Table 3).

## Discussion

### Principal findings

Day time primary care practices with more young children and foreigners in their patient populations had higher out-of-hours primary care use at the GP cooperative. In addition, shorter distance from the practice to the GP cooperative was associated with higher care use, indicating that easy accessibility of the GP cooperative stimulates out-of-hours care use. However, we hypothesised that aspects in the accessibility and organisation of day time primary care would also be associated with out-of-hours care use. We have found some evidence for this hypothesis. Patients used the out-of-hours GP cooperative less frequently when general practices had shorter telephone waiting times and when the GP was personally available for palliative patients. Several non-significant trends supported the idea that poor accessibility and availability during day time were associated with higher use of out-of-hours care, such as longer waiting times for non-urgent appointments, lower availability of a telephone consulting hour, lower availability for appointments after 5 p.m., and less frequent holiday openings.

Furthermore, the use of out-of-hours care services was higher in practices where GPs performed more diagnostic tests and therapeutic procedures. The high use group also had about 15% more contacts. The larger number of employment hours of practice assistants and higher experienced GP workload are congruent with this, as busy GPs try to reduce the workload with help from assistants. These findings may reflect worse patient health in the high use group. However, GP behaviour might play an important additional role: by performing a medical procedure, the GP confirms the necessity of the consultation and indirectly stimulates the patient to seek medical care in future, maintaining a vicious circle. Contrary to our expectations, educating patients was not related to out-of-hours primary care use, as in both groups a similar percentage of GPs talked to patients about unnecessary use of the GP cooperative.

### Strengths and weaknesses

Our sampling procedure resulted in two contrasting samples of general practices (high versus low use of out-of-hours care). This optimised the opportunities to identify relevant practice characteristics. Contrary to most other studies in this subject area, we did not use patient reports of accessibility, but we performed objective measurements (e.g. telephone accessibility) and used data from registration systems and from questionnaires/interviews with professionals. Due to the intensive data collection, we could only include 100 practices, which was a limitation for the statistical power of the study. We used validated measures of key factors and multivariate modeling, but we could not correct for all possibly relevant confounders. Because we had no data about health status, it remains unclear if the high utilisation practices have higher out-of-hours care use and, for example, higher numbers of tests and procedures because the health needs of the population make this justifiable. Moreover, a certain degree of significant results may be expected due to chance.

All data were gathered over the period of one year, except for the telephone accessibility measurement, which was performed up to 11 months later. There were no national interventions in telephone accessibility in that period and we believe accessibility will have remained largely stable over time in the practices.

When generalising the results to other countries, one has to bear in mind that the study was performed in a country with strong primary care. General practice is the first point of access of the healthcare system, nearly everybody is registered with a general practitioner, and general practice provides comprehensive medical care. The accessibility of general practices is high in the Netherlands, even in relatively low accessible practices. But still, the small absolute differences in accessibility between the practices (e.g. the difference in telephone waiting times was only one minute) had an effect on the use of out-of-hours acute care. The effect of accessibility improvement strategies will probably be larger in countries with weaker primary care systems.

### Comparison with existing literature

Other studies also found associations between patient characteristics and health care use. Like in our study, young children [8], foreigners [9,10], and patients living close to the service [12,14] were found to contact acute care services more often. Our findings regarding characteristics of the general practices related to out-of-hours care use are partly consistent with studies in the ED setting [9,16-23]. Similar to our results, Lowe et al. found that ED use was lower in practices with more evening hours. We were not able to confirm the positive associations found regarding the presence of nurse practitioners and regarding the ratio of patients to primary care clinician-hours [9].

Other studies into this subject were based on self-reports of patients. In line with our findings, they found that patients visiting the ED more likely perceived difficulties in getting telephone contact with the primary care provider [16,18,21,22] and in getting a timely appointment [16-18,20-23]. In contrast, Harris et al. did not find a relation between these and other perceived primary care access variables and ED use [19].

Finally, Giesen et al. examined motives of patients to contact a GP cooperative. For 21% of the respondents, poor accessibility of the general practice during office hours was an important reason for the out-of-hours contact [24].

### Implications for practice and research

Future studies should include intervention trials to explore the effects of changing practice characteristics on out-of-hours care use and to draw conclusions about causality. Moreover, the consequences of these interventions for the workload of GPs should be taken into consideration, since high utilisation practices already have a higher number of contacts per patient. It should also be examined if it is safe for patients to direct patient flows to day time general practices. Patients who are discouraged from seeking acute care may be placed at risk for adverse outcomes when they wait until the opening hours of the general practice. The health status of the patients might justify seeking contact with the GP cooperative. Finally, future research could include discussions with patients regarding the constraints they face. These conversations might provide new ideas that can be used to support care delivery improvements and might also contribute significantly to our knowledge about primary care delivery needs (patients) and patterns (staff and providers).

## Conclusions

Besides patient population characteristics, organisational characteristics of general practices are associated with lower use of out-of-hours care. Improving accessibility and availability of day time primary day care might be a potential effective way to improve the efficient use of out-of-hours care services.

## Abbreviations

GP: General practitioner
ED: Emergency department
CI: Confidence interval
Pt: Patient
